# Study of the tribological properties of surface structures using ultrashort laser pulses to reduce wear in endoprosthetics

**DOI:** 10.1186/s13018-020-01719-1

**Published:** 2020-06-03

**Authors:** Lea Theresa Backes, Paul Oldorf, Rigo Peters, Robert Wendlandt, Georg Schnell, Arndt-Peter Schulz

**Affiliations:** 1 Department of Orthopaedics and Traumatology/Laboratory for Biomechatronics, University Lübeck, Ratzeburger Allee 160, 23538 Lübeck, Germany; 2 SLV (Schweißtechnische Lehr- und Versuchsanstalt) Mecklenburg-Vorpommern GmbH (Gesellschaft mit beschränkter Haftung), Alter Hafen Süd 4, 18069 Rostock, Germany; 3 Department of Microfluidics, Faculty of Mechanical Engineering and Marine Technology, University of Rostock, Justus-von-Liebig Weg 6, 18059 Rostock, Germany; 4 Department of Trauma, Orthopaedics and Sports Medicine, Germany, BG (Berufsgenossenschaft) Klinikum Hamburg, Bergedorfer Strasse 10, 21033 Hamburg, Germany

**Keywords:** Aseptic loosening, Wear reduction, Ultrashort pulse laser, Microstructures, Surface structures, Endoprosthetics, Ring-on-disc test

## Abstract

**Background:**

Loosening of prostheses and functional disorders represent a far-reaching problem in the clinic, and the long-term outcomes are essentially determined by wear. Despite all advances, up to 10% of prostheses still fail after 10 years. In particular, more active patients show increased revision rates.

**Methods:**

The objective of this thesis is to examine whether the applied microstructures of the articulating surfaces can lead to a reduction in abrasion. Three different structural geometries (dimples, offset lines, grid lines) were defined. In an experimental test setup according to DIN ISO 6474 (Deutsches Institut für Normung, International Organization for Standardization), a tribological test of metal and ceramic pairings was performed using two-dimensional ring-on-disc (RoD) tests.

**Results:**

In both material groups, the structuring had a positive effect on the wear behaviour. In the ceramic group, an abrasion reduction of 22.6% was achieved. However, it is important to take into account the limited informative value due to the hardness of the material. Two of the three Cobalt-Chrome-Molybdenum (CoCrMo) structure geometries (grids, offset lines) also showed a significant reduction in abrasion compared to the reference group, with a maximum wear reduction of 55.5%.

**Conclusion:**

By reducing abrasion, surface structuring could be used to extend the life of prostheses and minimise the number of revisions.

## Background

In Germany, well over 400,000 joint replacement operations are performed annually. More than half of these operations (55.45%) are artificial hip replacements [1]. A further increase in endoprosthetic care can be expected in the future [2]. The demographic change of the population and the continuation of the trend towards a younger and more active group of patients mean that the demands are increasing not only in terms of functionality and resilience, but also in terms of the service life of the prostheses [3]. Despite all advances, up to 10% of prostheses still fail after 10 years. In particular, more active patients show increased revision rates [4]. In 2017, the proportion of follow-up procedures remained consistently high at over 61,000 operations (12.5%) [1]. The distribution is also reflected in the surveys of the German Endoprostheses Register. With almost 30,000 procedures, revision interventions were responsible for 10.4% of the recorded number of procedures [5].

The limited useful life may necessitate several replacement operations. Loosening of prostheses and functional disorders represent a far-reaching problem in the clinic, and the long-term outcomes are essentially determined by wear [3, 4]. Abrasive particles can cause foreign body reactions, lead to bone resorption and are considered the main cause of aseptic prosthesis loosening [6, 7].

The objective of this thesis is to examine whether the applied microstructures of the articulating surfaces can lead to a reduction in abrasion. A publication by Tarabolsi et al. addressed the micro-structuring of endoprosthetic surfaces by ultrashort laser pulses for the first time and demonstrated promising results [8]. By reducing wear and the associated aseptic loosening of the prosthesis, the service life of endoprostheses could be extended. By developing a reproducible and stable process for micromachining articulating surfaces with the aid of an ultrashort pulse laser, the structures could, if successful, also be transferred to endoprosthesis surfaces and thus contribute to the development of a reliable long-term solution. By reducing the number of revision procedures and subsequent complications, this could relieve the burden not only on patients, but also on the health system.

## Materials and methods

In cooperation with the SLV M-V GmbH, structuring was applied to the corresponding hard-hard friction pairings.

An ultrashort pulse laser (TruMicro 5 × 50, Trumpf Laser- und Systemtechnik GmbH & Co. KG, Ditzingen, Germany, pulse length 6 picoseconds), integrated in a high-precision micro-machining system GL.5 (GFH GmbH, Deggendorf, Germany), was used to generate microstructures on the articulating surfaces. With the help of this special laser technology, highly precise structures could be generated to realise a tribological functionalisation of the surfaces.

Based on previous experiences and tests, three different structural geometries were defined. The aim was to improve the rheological as well as tribological behaviour of the bearings and the low notch effect at the same time.

**Fig. 1:**
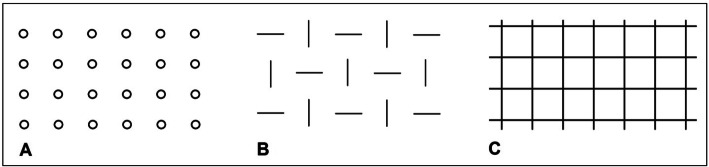
Defined microstructures (**a** dimples, **b** offset lines, and **c** grid lines)

All investigated microstructures are shown in Fig. 1. In the dimple pattern (A), conical holes with a diameter of about 20 μm were laser structured, which tapered conically to a depth of about 20 μm. The lateral distance between the dimples was 100 μm. With the offset lines (B), the groove structures were arranged separately and not connected to each other. The structures had a depth and width of 20 μm and were slightly conical. The length and distance of the trenches were each 100 μm. The parallel grid pattern (C) showed slightly conical lines with a width and depth of around 20 μm. The lateral distance of the grid lines was 100 μm.

In an experimental test setup according to DIN ISO 6474 [9], a tribological test of the differently structured metal (CrCoMo, OHST Medizintechnik AG, Rathenow, Germany) and ceramic (ELEC®*plus*, HiPer Medical AG, Oberkrämer, Germany) pairings was performed using two-dimensional ring-on-disc (RoD) tests (Tribometer TRM 1000, Company Wazau, Berlin, Germany). Each group had five test samples with a determined geometry. The test discs were provided with circular defined microstructures; the rings remained unstructured. Unstructured sample pairs served as reference samples (Table 1).

**Table 1:** Trial design ring-on-disc

**Pairings**	**Structure**	**Width** **[μm]**	**Depth** **[μm]**	**Number [pieces]**
ELEC®*plus*	Reference	–	–	5
ELEC®*plus*	Dimples	20	20	5
ELEC®*plus*	Offset lines	20	20	5
ELEC®*plus*	Grid lines	20	20	5
CoCrMo	Reference	–	–	5
CoCrMo	Dimples	20	20	5
CoCrMo	Offset lines	20	20	5
CoCrMo	Grid lines	20	20	5

The specimens were produced with a fixed geometry: the disc had a diameter of 25 mm, the ring had an outer diameter of 20 mm and an inner diameter of 14 mm. The ring was formed by a 2-mm countersunk hole in such a way that the contact surface of the two components resulted from the difference between the outer and inner radius of the ring. The test was carried out with uniform pressure distribution and regular changes in the direction of movement with interruption of the lubricating film. The test was performed over a period of 100 h with an axial force of 1500 N, and the temperature was 37 °C. The ring rotated with an angle of ± 25° at a frequency of 1 Hz on the fixed disc. The change in the direction of movement was approximately sinusoidal. The roughness of the samples used was set to ≤ 0.10 μm, and the flatness was set to ≤ 0.6 μm. At the end of the test, the specimens were cleaned in an ultrasonic bath with distilled water for a total of 33 min [9].

**Fig. 2:**
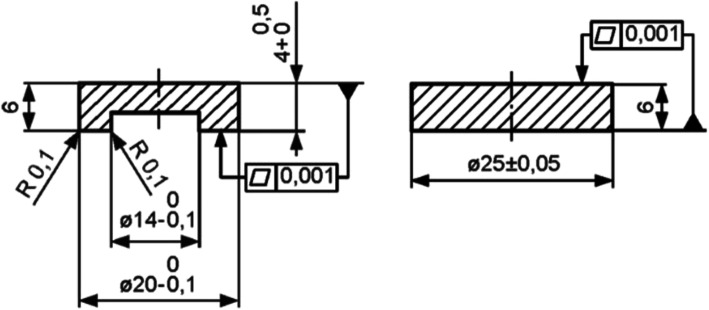
Technical drawing of the RoD test specimens according to ISO 6474:1994 [9]

To decrease the friction torque, diluted calf serum was used as a replacement for natural synovial fluid. In accordance with the norm DIN ISO 6474, 25% of this was dissolved in distilled water. The additives ethylenediaminetetraacetic acid (EDTA) and Patricin were used to stabilise the pH value and prevent fungal or bacterial contamination (Fig. 2) (Table 2).

**Table 2:** Composition of the applied joint replacement fluid

**Lubricant**	**Protein content [g/l]**	**EDTA [g/l]**	**Patricin [ml/1, 5 l]**
Diluted calf serum	20	8	0, 1

A digital light microscope “VHX-600” (Keyence, Osaka, Japan) was used to examine the structural results. The optical analysis and evaluation of the test results was performed using a confocal laser scanning microscope “VK-X200” (optical zoom 1 to × 8, total magnification 200 to × 24000; Keyence, Osaka, Japan) and the “VK analysis software” (Version 3.3.0.0.). When evaluating the raw data, the data was first sorted and displayed in Excel Version 14.4.8. The statistical evaluation was carried out using Minitab Version 18.1., R Version 3.5.1 and RStudio Version 1.1.463.

The measured variable of this research was material removal depth. To determine this value, the surface of the disc was divided into six equidistant positions at an angle of 60° (measuring fields 0°, 60°, 120°, 180°, 240°, 300°). At each of these positions, the profile height difference as compared to the initial state was determined in measuring fields with a length of about 3 mm, which were assembled by stitching 20 single measuring fields along the measuring length by using a long-distance lens with a × 50 magnification. All six measured values per specimen were then averaged. The median was used for significance testing due to its robustness. Particularly with small data sets, outliers strongly influence mean values, thus the decision was made to use median values.

## Results

**Fig. 3:**
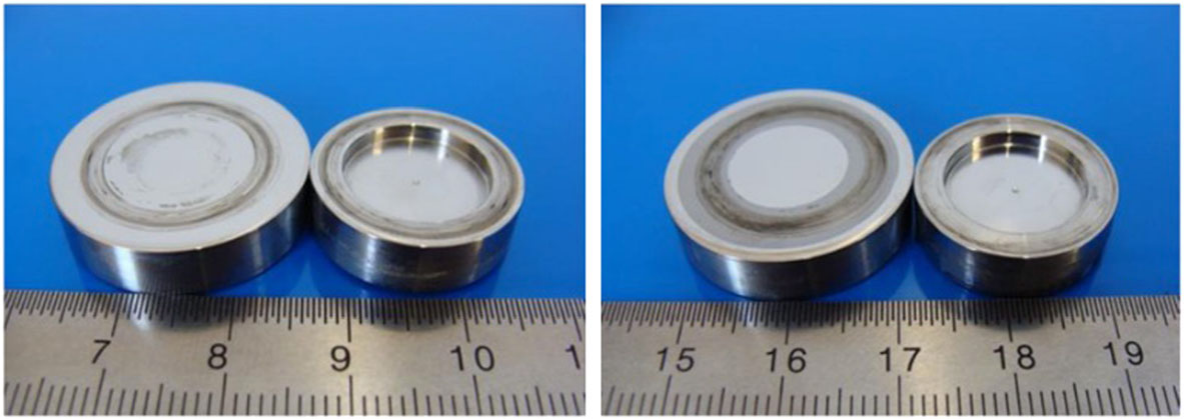
Ring-on-disc test specimens made of CoCrMo after the test procedure (left: unstructured, right: grid line structure)

**Fig. 4:**
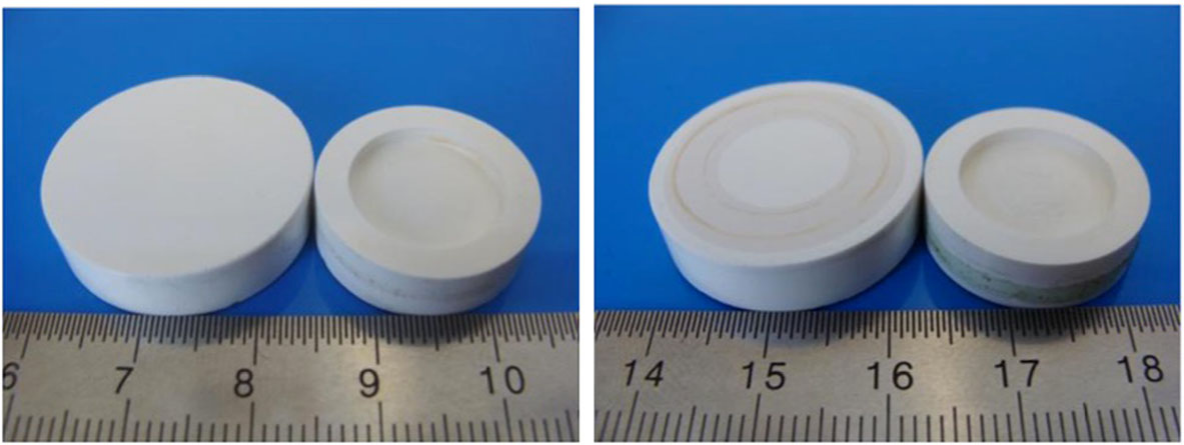
Ring-on-disc test specimens made of ELEC®*plus* after the test procedure (left: unstructured, right: grid line structure)

Figure 3 shows two CoCrMo specimen pairs after the ring-on-disc test. Wear-related friction traces and serum residues are already visible. Figure 4 shows two ELEC®*plus* specimen pairs after 100 h of ring-on-disc testing.

Due to the extreme hardness of the ceramic components, only minimal abrasion could be measured in both groups (reference, grid lines) using confocal microscopy. The structured ELEC®*plus* grid lines group had, with 0.0986 μm, a slightly lower wear compared to the unstructured reference group (0.1274 μm). The median of the grid lines group was 0.0803 μm, and the median of the reference group was 0.1108 μm.

A non-parametric method was used for the significance test. The Wilcoxon test showed a significant wear reduction due to the usage of grid lines (*p* value 0.03175). Despite this result, we decided against further testing of the two remaining structural geometries (dimples, offset lines), as the wear was very low in both test groups even after 100 h of testing due to the extreme hardness of the material (Table 3).

**Table 3:** Removal depth [μm] ELEC®*plus* specimen pairs after RoD test

	**Structure**	**Mean**	**StDev**	**Min**	**Q1**	**Median**	**Q3**	**Max**
Depth removal [μm]	Reference	0.1274	0.0601	0.0509	0.0829	0.1108	0.1661	0.2856
	Grid lines	0.0986	0.0567	0.0333	0.0593	0.0803	0.1257	0.2461

*StDev* standard deviation, *Min* minimum, *Q1* first quartile, *Q3* third quartile, *Max* maximum

**Fig. 5:**
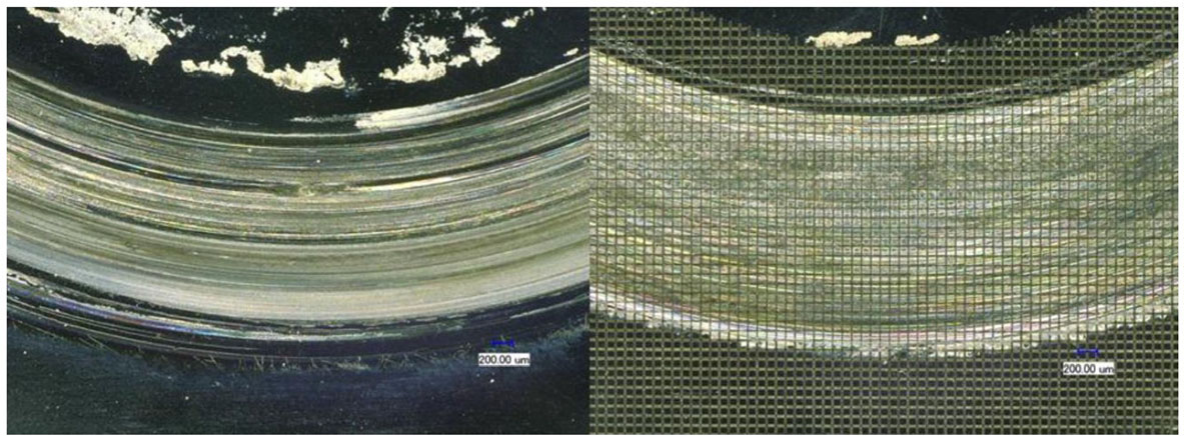
Light microscope images of the CoCrMo samples after the ring-on-disc tests (left: unstructured, right: gridline structure)

The CoCrMo test specimens were tested as planned. A significant abrasion reduction through micro-structuring was observed. Figure 5 shows deep scratches on the reference specimen after the test.

**Fig. 6:**
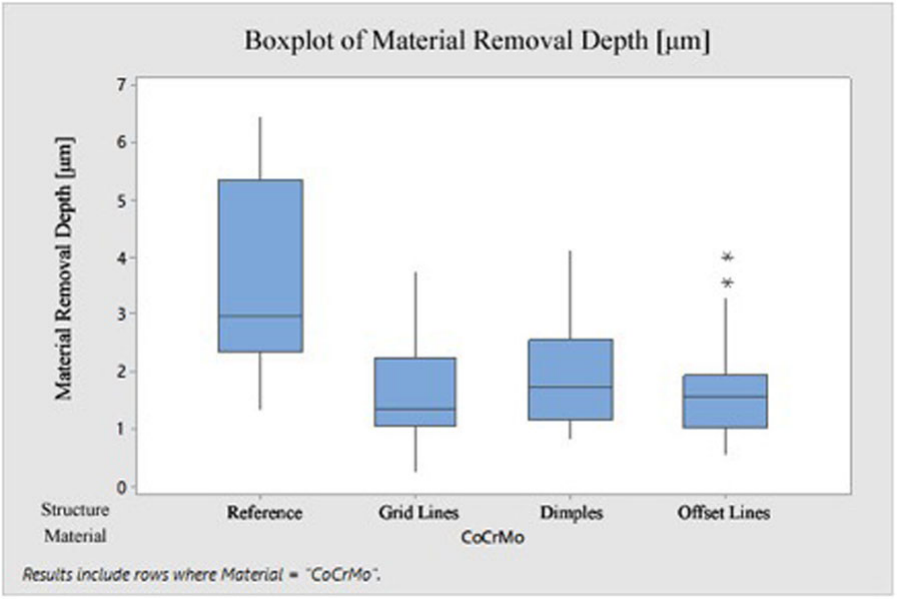
Boxplot material removal depth-CoCrMo sample pairs after RoD test

The results of the CoCrMo specimens can be seen in Fig. 6. The Kruskal-Wallis test and the pairwise post-hoc test were used for significance testing. The *p* value correction was performed using the Benjamini-Hochberg method.

The unstructured reference group showed the highest average material removal depth with a value of 3.691 μm; the median was 2.950 μm. All applied structures led to a reduction in abrasion to varying degrees. In descending order, dimple patterns followed with 1.996 μm (median 1.722 μm), offset lines with 1.662 μm (median 1.565 μm) and grid patterns with 1.643 μm (median 1.337 μm). When comparing the measurement results of the various patterns applied, it became apparent that the dimple group had the highest wear rates. The other two groups (grid, offset lines) differed only slightly in their abrasion. The grid patterns (*p* value = 0.0261) and the offset lines (*p* value = 0.0207) led to a significant wear reduction. The differences in wear reduction between the two individual geometrical variants, however, were not proven to be statistically significant (Table 4).

**Table 4:** Removal depth [μm] CoCrMo specimen pairs after RoD test

	Structure	Mean	StDev	Min	Q1	Median	Q3	Max
Depth removal [μm]	Reference	3.691	1.681	1.344	2.354	2.950	5.368	6.435
	Grid lines	1.643	0.863	0.273	1.046	1.337	2.247	3.746
	Dimples	1.996	0.960	0.835	1.169	1.722	2.552	4.125
	Offset lines	1,662	0,833	0,545	1,019	1,565	1,938	3,995

*StDev* standard deviation, *Min* minimum, *Q1* first quartile, *Q3* third quartile, *Max* maximum

## Discussion

Continued growth in endoprosthetic care can be expected in the future. Due to the limited service life and the steadily increasing number of implants, there is a need for reliable long-term solutions. Aseptic prosthesis loosening continues to be the leading cause of revision [5, 10].

The surface structuring of the sliding pairing offers a new starting point for reducing aseptic prosthetic loosening. The first promising research took place in 2011 which examined patterned CoCrMo and Al2O3 surfaces for reduced free wear debris in artificial joint arthroplasty [8].

The results of our ring-on-disc tests confirm that an improvement in abrasion behaviour can be achieved by micro-structuring the surfaces. In both material groups, the structuring had a positive effect on the wear behaviour. A significant reduction in abrasion was achieved. This result could also lead to a possible significant reduction in wear in vivo. We could see that the differently structured patterns had an effect on the measured abrasion quantity. The gridlines were the most promising pattern, whereas the dimple structure was the least promising.

There are several research projects dealing with the micro-structuring of surfaces of different materials to reduce abrasion of endoprostheses. In particular, the effect on the lubricant film thickness and the coefficient of friction has been investigated. As a result, friction and wear were reduced [11, 12]. Furthermore, micro-structuring has an effect on rheological properties. According to Myant et al., the fluid undergoes bulk phase separation rheology during sliding. This results in an increased protein phase in less or unstressed areas, which forms a protective hydrogel film [13]. Local rheological changes and the aggregation of proteins of the synovial fluid influence the formation of interfacial film. Local phase changes were observed with a strongly increased protein content and a much higher viscosity than the rest of the fluid. This enabled a thicker lubricating film at lower shear rates [14]. A study investigated the influence of surface functionalization on viscosity of the synovial fluid. An increase in viscosity could be demonstrated by micro-structuring. This could lead to better hydrodynamic lubrication and lower particle abrasion [15]. These findings could also influence the tribological performance of endoprostheses in the future.

However, surface modification not only serves to reduce friction between the sliding partners but should also improve bone integration. Experiments to improve bone integration are based on surface and porosity changes and on various coatings. The shaft component of endoprostheses has been the focus of much research in the past to create early bone integration, especially in cement-less endoprostheses [16]. The surface modification of implants by means of plasma coating (plasma-spray, plasma polymerisation) is still of great importance. This process is used to produce partially or completely melted substrates with which apatite, metal, ceramic or polymer layers can be applied to the substrate. This can influence biocompatibility and bioactivity [17]. A review published in 2018 described the latest advances in ionic substitution in thin hydroxyapatite films. Clinical results of traditional hydroxyapatite coatings did not achieve the desired results. Similarly, plasma spray coatings have a tendency to crack and delaminate due to their relatively high thickness. For this reason, research in recent years has focussed on two topics in particular: the investigation of ion-substituted apatites and the development of plasma-assisted techniques for the deposition of thin films [18].

In addition to micro-structuring, there are numerous other efforts to reduce the number of revision procedures and increase the service life of endoprostheses. New research is investigating ways to reduce endoprosthetic loosening at the molecular level. By inhibiting osteoclast activity, bone loss is minimised. In vitro studies have shown that osteoclast activity can be blocked by the use of denusomab, a human monoclonal antibody (IgG2 anti-Receptor Activator of NF-κB-Ligand/RANKL antibody). Based on these results, a study protocol was prepared for a randomised, double-blind, placebo-controlled study. However, this study is not yet complete and continues to recruit patients [19]. Another suppressor of RANKL-stimulated osteoclast activity was found in Jatrorrhizine hydrochlorides. It is an alkaloid isolated from a traditional Chinese plant (Coptis chinensis). These molecular findings could also lead to a novel therapeutic natural agent for the treatment of periprosthetic osteolysis in the future [20].

Another important point is the head size. A review published in 2018 dealt with the head size factor in hip endoprostheses and its effects on luxation risk and abrasion behaviour across several various countries [10]. In the last decade, the trend has shifted towards the use of larger endoprosthesis heads. The use of large femoral heads appears to be a compromise between increased stability and reduced survival of the prosthesis. Head sizes above 36 mm do not seem to have any advantage in terms of the range of motion and hip function. Today, 32 mm and 36 mm head diameters are mainly used. However, the increased friction surface must be taken into account because both volumetric wear and friction torque increase with increasing size [21].

However, the ring-on-disc method represents a two-dimensional, standard-compliant simulation method for wear testing. Of course, these tests do not reflect the complexity of the actual stress processes in the human body in all completeness. Therefore, the evaluation of the results must take into account the limited informative value. A direct transfer of the results to the behaviour of the materials in vivo is not possible.

In addition, ultrashort pulse laser technology is also subject to economic limits. The high peak power and extremely short interaction times of this laser make efficient processing of materials possible practically without thermal or mechanical influence. However, the structuring of large surfaces or the generation of structures with high roughness not only increases the processing time, but also the manufacturing costs.

Furthermore, it would be important to investigate the behaviour of such micro-structured surfaces with other material pairings such as UHMWPE-on-Ceramics, which are more commonly used but have a lower wear resistance compared to hard-on-hard bearing pairs.

## Conclusion

The limited service life of prostheses presents society with a major challenge not only from a medical but also from an economic point of view. The main reason for revision interventions is wear-induced aseptic loosening. Abrasion occurs at all interfaces of the prosthesis components, but above all between the sliding partners themselves.

Two-dimensional ring-on-disc investigations were performed to investigate various structural geometries on metal and ceramics. We were able to show that in the area of the CoCrMo surfaces, a clinically possible significant wear reduction can be achieved. The dimple structure was the least promising pattern.

Due to the extreme hardness of the ceramic components, an extension of the test duration or an increase in the number of samples would have been necessary for further testing of this material. However, since this test setup would not have resulted in a clinically significant reduction in abrasion, we decided against further testing. The question of whether laser processing could additionally influence the problem of squeaking of ceramic pairings might be of interest for the future.

By reducing abrasion, surface structuring could be used to extend the life of prostheses and minimise the number of revisions. This would not only save patients from early revision procedures but would also provide economic relief for society as a whole.

## Data Availability

The datasets during and/or analysed during the current study are available from the corresponding author on reasonable request.

## Abbreviations

SLV M-V: Schweißtechnische Lehr- und Versuchsanstalt Mecklenburg-Vorpommern
GmbH: Gesellschaft mit beschränkter Haftung Limited liability company
BG: Berufsgenossenschaft (Institution for Statutory Accident Insurance and Prevention)
DIN ISO: Deutsches Institut für Normung (German Institute for Norms) International Organization for Standardization
RoD: Ring-on-disc
CoCrMo: Cobalt-Chrome-Molybdenum
EDTA: Ethylenediaminetetraacetic acid
Al2O3: Aluminium oxide
RANKL: Receptor Activator of NF-κB Ligand
UHMWPE: Ultra-high-molecular-weight polyethylene
